# Compliance with Physician Follow-up Appointments After Acute Traumatic Hand and Forearm Injuries: A Prospective Comparison of Demographic, Socioeconomic, and Clinical Characteristics

**DOI:** 10.5152/eurasianjmed.2026.251324

**Published:** 2026-03-09

**Authors:** İremnur Özcan Özel, Hande Özdemir, Erol Benlier

**Affiliations:** 1 Department of Physical Medicine and Rehabilitation, Trakya University Faculty of Medicine, Edirne, Türkiye; 2 Department of Plastic, Reconstructive and Aesthetic Surgery, Trakya University Faculty of Medicine, Edirne, Türkiye

**Keywords:** Appointments, hand injuries, patient compliance

## Abstract

**Background:**

The aim was to compare the demographic, socioeconomic, and clinical characteristics of patients who were compliant with and non-compliant with physician follow-up appointments at a hand rehabilitation clinic after acute traumatic hand and forearm injuries.

**Methods:**

The study included 81 patients with hand and forearm injuries. At the initial presentation, patients’ demographic and socioeconomic characteristics, nature and severity of injury, scores of Brief Illness Perception Questionnaire, Hospital Anxiety and Depression Scale, and Exercise Benefits/Barriers Scale, previous injury history, and rehabilitation experiences were recorded. Permanent workers were also questioned about the urgency of returning to work and the impact of the injury on their ability to continue working. Those who attended all 6 follow-up appointments (compliants) and those who did not attend at least 1 follow-up appointment (non-compliants) were compared on variables recorded.

**Results:**

The study included 62 males (76.5%) and 19 females (23.5%), aged 18-76 years, with a median age of 31 years. No significant differences were found between compliant and noncompliant patients in terms of demographic and socioeconomic factors, injury-related clinical features, illness perception, psychological status, exercise benefit/barrier perceptions, previous injuries, and perceived return-to-work urgency. Patients who reported being unable to work without using their injured hands, who received hospital-based outpatient rehabilitation, and who had prior rehabilitation experience were more likely to attend follow-up appointments.

**Conclusion:**

The study confirmed the concept that patient compliance with physician follow-up appointments in patients with hand and forearm injuries constitutes a multifaceted health behavior.

**Main Points**

- Individuals who are compliant or noncompliant with scheduled physician appointments at hand rehabilitation clinics after acute traumatic hand or forearm injuries do not exhibit significant differences in terms of personal beliefs or traditional demographic and socioeconomic indicators.
- A salient factor distinguishing the compliant and noncompliant workers pertained to the significance workers ascribed to the repercussions of injury on their capacity to persist in their work.
- Higher prevalence of hospital-based rehabilitation among compliant patients compared to the noncompliant cohort.
- Patients with a history of prior rehabilitation exhibit higher compliance rates with follow-up appointments.

## Introduction

The hand and forearm are frequently the sites of musculoskeletal injuries, which can have a significant impact on an individual's daily activities and work capabilities.^1^ Young and economically active individuals are primarily affected, leading to significant economic strains on healthcare systems.^2^ Optimal treatment outcomes often require intensive postoperative care, particularly in cases necessitating surgical interventions.^3^

The shift from using “compliance” to “adherence” in healthcare terminology reflects a focus on encouraging patient engagement rather than implying a passive role.^4^ Treatment compliance involves various behaviors, such as home exercises, therapy appointments, and following healthcare provider recommendations.^5^ Failure to maintain consistent attendance at medical appointments in outpatient clinics can result in various adverse consequences, including the disruption of meticulous planning, a decline in the quality of care, the misapplication of resources, a decline in patient satisfaction, and the interruption of scientific research processes.^6^ Several studies examining the characteristics of patients with upper extremity trauma who do not attend physician appointments have shown that these patients tend to be male, younger, non-white, unmarried, living far from the health care facility, and socioeconomically disadvantaged.^6^^-^^9^ Prospective studies are essential for gathering detailed information, understanding the impact of compliance on clinical outcomes, and developing interventions to improve follow-up attendance early on in injury for successful recovery.^10^ The objective of this study is to conduct a comparative analysis of the demographic, socioeconomic, and clinical characteristics of individuals who are compliant and non-compliant with physician controls in a hand rehabilitation clinic following acute hand and forearm injuries through prospective observation.

## Material and Methods

### Participants

Between January 2024 and January 2025, 95 patients with acute traumatic hand and forearm injuries who underwent surgical treatment at Trakya University Faculty of Medicine Hospital and who were literate in Turkish were screened for eligibility for the study. Following the exclusion of subjects under the age of 18 and those with psychological or mental disorders that would prevent them from understanding the questions, a total of 81 patients were included in the study. All participants signed the informed consent form, agreeing to participate in the study according to the Helsinki Declaration.

### Evaluations

A comprehensive evaluation of the participants was conducted during their initial hospital visit, encompassing various demographic and socioeconomic factors. The factors considered included gender, age, educational attainment, marital status, living arrangement, providing for the family, employment status, and the number of working days per week and hours per day. The monthly income, social security status, alcohol and tobacco use, vehicle ownership and driver’s license status, access to public transportation, method of reaching the hospital, and geographical distance from residence (in kilometers and minutes) were also considered. The geographical distance from residence was calculated by Google Maps. The evaluation also included an inquiry into whether someone provided care at home and whether the subjects were responsible for caring for someone else.

Participants were also evaluated based on clinical characteristics. The participants were asked to respond to inquiries regarding the dominance of the injured limb and whether the injury resulted from a work-related accident. Excluding seasonal workers, permanent workers were asked to choose one of the following options in terms of the urgency of returning to work: urgent, somewhat urgent, and not urgent. In addition, the subjects were presented with the following inquiry: “To what extent does the injury impact your ability to continue your work?” The options ranged from “Very important; it is impossible for me to continue my work without using my injured hand” to “Important; it becomes very difficult for me to continue my work,” “Not very important; I can partially continue my work,” and “Not important; I can perform my work well despite the injury.” The nature and severity of the injury sustained were ascertained and meticulously documented. The severity of the injury was assessed using the Modified Hand Injury Severity Score (MHISS). According to MHISS, injuries were classified as mild (20 points or less), moderate (21-50 points), severe (51-100 points), and major (101 points or more).^11^^,^^12^

In order to assess their emotional and cognitive representations, including outcomes related to their illness, timeline, personal control, treatment control, identity, coherence, concern, emotional response, and causes, participants were administered the Turkish adaptation of the Brief Illness Perception Questionnaire (BIPQ). Higher scores indicated a greater perception of illness.^13^^,^^14^ The Hospital Anxiety and Depression (HAD) Scale, which has been adapted into Turkish, was utilized to assess anxiety and depression in patients. Participants whose total scores for items 1, 3, 5, 7, 9, 11, and 13, i.e., the HAD-Anxiety score, exceeded 10 points were considered at risk for anxiety. Participants whose total scores for items 2, 4, 6, 8, 10, 12, and 14, i.e., the HAD-Depression score, were above 7 were considered at risk for depression.^15^^,^^16^ The participants were asked whether they had previously sustained an injury in the same hand or forearm and whether they had undergone physical therapy for an injury. The participants’ responses were documented. The Turkish version of the Exercise Benefits/Barriers Scale (EBBS), which was developed by Sechrist et al,^17^ was utilized to assess the perceived benefits and barriers of exercise. The total score from the 29 benefit items was documented as the perceived benefit, and the total score from the 14 barrier items was documented as the perceived barrier level.^18^ During the initial assessment, patients were informed that they were required to attend physician’s appointments at the hand rehabilitation outpatient clinic on a biweekly basis for a period of 12 weeks. All patients were given general recommendations during the initial consultation regarding wound care, swelling management, scar massage, and orthotic use. Appropriate therapeutic exercises were recommended and described according to the type of injury. All patients were then offered a hospital-based rehabilitation program. Those who accepted the hospital-based rehabilitation program were enrolled in a 6-week program, 3 days a week, starting from the 3rd week after the injury. Patients who declined to participate in a hospital-based rehabilitation program were offered a home-based exercise program. The number of outpatient clinic visits that patients attended was recorded. Individuals who attended all 6 follow-up appointments within the 12-week period were classified as compliant, while those who missed any follow-up appointments were considered non-compliant.

### Sample Size Calculation

Based on the study conducted by Bennet et al^19^ a power analysis was performed. An effect size of 0.31 was calculated, and it was determined that a total of 81 participants needed to be included in the study to achieve 80% statistical power at a 5% significance level.

### Analysis of Data

Data were analyzed using the Windows SPSS 23.0 software (IBM, Chicago, IL, USA). The normal distribution of continuous variables was assessed using the Shapiro–Wilk test. Descriptive statistics for normally distributed continuous variables are presented as the mean ± standard deviation, while non-normally distributed continuous variables are presented as the median (minimum-maximum). Categorical variables are presented as the number and percentage (n,%). For comparative statistics between the 2 groups, the independent samples *t*-test was used for normally distributed continuous variables, and the Mann–Whitney *U-*test was used for non-normally distributed continuous variables. Categorical variables were compared using the Pearson chi-square test or Fisher’s exact test, where appropriate. All results were evaluated at a significance level of *P* < .05.

## Results

The participants included in the study consisted of 62 (76.5%) males and 19 (23.5%) females, ranging in age from 18 to 76, with a median age of 31 years. The patient education levels indicated that 21.0% of the sample had a primary school education, 17.3% had a secondary school education, 46.9% had a high school education, 13.6% had a bachelor's degree, and 1.2% had a master’s degree. The married status of the patient population is as follows: 50.6% of patients were married, 44.4% were single, and 4.9% were divorced. The evaluation of patients according to their employment status revealed that 11.1% of the sample were unemployed, 6.2% were housewives, 37% were workers, 2.5% were civil servants, 18.5% were self-employed, 12.3% were retired, and 12.3% were in other employment groups. In evaluating the monthly income of the participants, it was determined that 29.6% of the participants reported an income below 10,000 Turkish Lira (TL), 51.9% between 10,000 and 30,000 TL, 14.8% between 30,000 and 75,000 TL, and 3.7% above 75,000 TL. The study found that 56.8% of patients were current smokers, while 43.2% reported not smoking. The study found that 43.2% of patients consumed alcohol, while 56.8% reported abstaining from alcohol. In the present study, 59% of patients sustained the current injury to their dominant hand, while 41% experienced the injury to their non-dominant hand. A total of 18.5% of the injuries were attributed to accidents in the workplace, and 34.6% of patients reported a previous injury to the hand and forearm on the same side. Preliminary research indicates that 9.9% of all injuries are attributable to instances of punching the window. The majority of the injury types (30.9%) consisted of combined injuries involving various combinations of tendons, arteries, nerves, and fractures. Subsequent to this, isolated tendon injuries (28.4%) and isolated fractures (25.9%) were documented. The study found that 7.4% of the sample had amputations, 3.7% had crush injuries, 2.5% had isolated nerve injuries, and 1.2% had lacerations. Of the fractures, 18 were phalangeal fractures, 6 were metacarpal fractures, and 5 were distal radius fractures. The spectrum of nerve injuries included 9 digital nerve injuries, 5 median nerve injuries, 2 ulnar nerve injuries, 1 radial nerve injury, 1 median and ulnar nerve injury, and 1 median and radial nerve injury. While 43.2% of participants were observed to be compliant with follow-up appointments, 56.8% were observed to be non-compliant. When patients who were compliant with and non-compliant with follow-up appointments were compared in terms of demographic and socioeconomic characteristics, no significant difference was found between the 2 groups (Table 1).

The comparative analysis of permanent workers revealed no significant difference between the compliant (n = 24) and non-compliant (n = 28) workers regarding their perceived urgency to return to work (*P* = .893). However, a statistically significant difference was observed in how the groups perceived the injury's impact on their work ability (*P* = .029). Notably, a higher proportion of compliant workers (66.7%) reported that it was impossible to continue their work without using the injured hand, compared to 53.6% in the non-compliant group. Detailed responses regarding work urgency and injury impact are presented in Table 2.

The groups compliant with and non-compliant with follow-up appointments were compared in terms of clinical features related to the type and severity of injury, illness perception, psychological status, exercise benefit and barrier perceptions, history of previous injuries and rehabilitation experiences, and the rehabilitation process. The findings of this analysis are delineated in Table 3.

## Discussion

In this study, demographic, socioeconomic and clinical characteristics of patients with acute traumatic hand and forearm injuries who were compliant with and non-compliant with physician appointments in a hand rehabilitation outpatient clinic were compared. Patients who stated that it was impossible to continue working without using their injured hand, who were enrolled in a hospital-based outpatient treatment program for their current injury, and who had previously undergone rehabilitation due to an injury were more likely to be in the compliant group for follow-up appointments. Conversely, patients who asserted that they could adequately perform their job despite their hand injury, who were undergoing a home-based treatment program for their current injury, and who had not previously engaged in a physical therapy and rehabilitation program were more likely to be in the non-compliant group for follow-up appointments. No statistically significant differences were found in the assessed demographic and socioeconomic characteristics, other clinical characteristics related to the current injury, disease perception, psychological state, perceived benefits, and limitations of exercise, or previous injury experiences.

Numerous studies in the literature have examined the effects of demographic and socioeconomic characteristics on follow-up and treatment compliance in patients with hand and forearm injuries.^7^^-^^9^^,^^20^^-^^25^ The extant literature offers equivocal results regarding the relationship between demographic factors and the outcomes under consideration. With respect to age, while it has been observed that younger patients^6^^,^^8^^,^^26^ may exhibit higher rates of noncompliance, certain studies^23^^,^^24^ have identified no significant correlation between age and compliance. While some studies^8^^,^^9^^,^^22^ have identified a correlation between male gender and non-compliance, other studies have failed to replicate this association.^7^ Level of education (i.e., lower education level) and marital status (i.e., being single or divorced) have been associated with non-compliance in some studies.^6^^,^^7^^,^^23^ It has been identified by some authors that practical barriers and logistical issues are significant factors that may influence compliance, independent of sociodemographic factors. Transportation difficulties, such as distance to the clinic and lack of driver's license, have been reported as reasons for non-attendance.^8^^,^^9^^,21^ Socioeconomic characteristics have been shown in some studies to influence compliance to follow-up and treatment in patients with hand and forearm injuries. Specifically, insurance status has been identified as a prominent determinant. Patients with or without Medicaid insurance have been reported to have significantly lower rates of attendance at physical therapy appointments and a higher likelihood of no-shows compared with patients with private insurance.^7^^-^^9^^,^^20^^,^^23^ Concurrently, the literature has identified a correlation between non-compliance and factors such as unemployment, disability, low income, and low socioeconomic status.^7^^,^^20^^,^^23^^,^^25^ These findings contradict the lack of sociodemographic differences observed in the study between compliant and non-compliant groups for follow-up appointments. This absence of sociodemographic influence may be attributed to the homogeneity of the single-center cohort and the relatively uniform healthcare access provided at the facility, which likely mitigated the impact of insurance or income status.

Depression, which has been linked to socioeconomic status, has been identified as a significant predictor specifically for orthosis compliance in patients with hand injuries.^27^ However, the current study—which evaluates compliance based on attendance at follow-up appointments—found no significant differences in psychological status between the compliant and noncompliant groups. This discrepancy suggests that psychological factors might influence different aspects of compliance (e.g., device use versus clinic attendance) in distinct ways. The extant literature defines compliance through various parameters, such as orthosis use,^22^^,27^ medical appointment attendance,^21^ or home exercise fidelity.^28^ The utilization of disparate definitions complicates the comparison of compliance rates and associated factors. Consequently, the findings suggest that strategies to enhance compliance should be broadly formulated for all patient demographics, as traditional demographic, socioeconomic, and psychological profiles may not universally predict follow-up attendance.

It is noteworthy that no statistically significant difference was found in this study between individuals with hand and forearm injuries who were compliant with and non-compliant with follow-up appointments in terms of either BIPQ or EBBS scores. This finding indicates that an individual's internal perceptions of illness and exercise alone are inadequate for determining compliance with the rehabilitation process.

While a significant proportion of permanent workers in both groups (35.7% to 41.7%) perceived return to work as "urgent," this comparable distribution in perceived urgency indicates that pressure to return to work alone is not a factor determining compliance to follow-up appointments. In summary, while both compliant and non-compliant workers perceived a comparable level of urgency, their compliance behaviors exhibited distinct differences.

The majority of workers who were compliant with follow-up appointments (66.7%) attested to the substantial impact of the injury on their capacity to continue working, with many reporting that it rendered their ability to do so impossible. In the noncompliant group, the proportion of respondents who indicated that the injury had a "Not very important" impact on their work (32.1%) was significantly higher than in the compliant group (4.2%). This finding suggests that individuals who perceived the injury's impact on functional ability to be more severe were more likely to comply with follow-up appointments. It is possible that compliant workers have recognized that their injury directly impeded their ability to perform their work and have viewed rehabilitation as an essential step in overcoming this obstacle. In contrast, workers in the noncompliant group, who perceived the injury's impact less acutely, may not have recognized the urgency or necessity of rehabilitation. This analysis indicates that the perceived severity of the injury on individual functioning, rather than external time pressure, plays a statistically significant role in compliance with follow-up appointments.

The present study demonstrates that hospital-based rehabilitation programs and prior experiences with physiotherapy are associated with compliance with physical therapy and rehabilitation outpatient clinic visits. The findings underscore the significance of continuous supervision, prompt feedback, motivational support, and professional guidance in the rehabilitation process of patients with upper extremity injuries.^29^ This controlled environment may have increased compliance by ensuring patients perform the exercises correctly, while also creating an atmosphere of social responsibility and expectation.

While home-based programs do offer a certain degree of flexibility, they also necessitate a higher level of intrinsic motivation and self-efficacy from the participants. The paucity of supervision and the presence of potential distractions have been posited as contributing factors to suboptimal compliance in these programs.^30^ Additionally, the observation that patients participating in the home-based exercise program were not necessarily those who accepted the hospital-based program suggests that these patients may already have barriers to regular hospital visits due to personal or environmental reasons. Conversely, prior experience with physiotherapy may have facilitated the development of a positive schema regarding the rehabilitation process, thereby reinforcing a positive expectancy-outcome relationship. A plethora of studies have examined the role of motivation, self-efficacy, self-identity, and habit strength in the initiation and maintenance of health behaviors.^31^^,^^32^ Taken together, these findings suggest that scales assessing an individual's psychological state, beliefs about illness and exercise alone are insufficient in predicting compliance to follow-up appointments during the rehabilitation process. Instead, the nature of the exercise program (hospital-based vs. home-based) and the individual's previous physiotherapy experience may play an important, interactive role in compliance.

There are some limitations that should be considered when interpreting the results of this study. The limitations of the study include its exclusive focus on a single center, which may restrict the generalizability of its findings to other geographic populations. It is important to note that similar studies conducted in populations residing in regions with varying characteristics, such as transportation infrastructure and sociodemographic composition, may yield distinct results regarding compliance factors. Moreover, the exclusive focus of the study on a particular group of patients with hand and forearm injuries serves to constrain the extent to which its findings can be extrapolated to other types of injuries or medical conditions. In light of these limitations, the execution of multicenter studies, which would entail the collection of data from disparate geographic and socioeconomic regions, would prove advantageous in fostering a more comprehensive understanding of the factors that influence compliance with follow-up. To enhance the generalizability of the findings, it is recommended that studies be conducted with patient populations exhibiting diverse musculoskeletal injuries or chronic conditions.

The present study corroborated the notion that patient compliance with physician follow-up appointments constitutes a multifaceted health behavior in patients with hand and forearm injuries. The findings indicated that individuals who were compliant with their appointments differed not in terms of demographics, socioeconomic status, psychological status, and internal perceptions, but rather in terms of the perceived functional severity of the injury regarding work ability, hospital-based rehabilitation involvement, and previous physiotherapy experience. From a clinical perspective, the findings support the idea that strategies aimed at enhancing compliance in patients with acute traumatic hand and forearm injuries should prioritize education that emphasizes the profound functional impacts of the injury and direct patients to the implementation of structured, clinical-based programs. The formulation of strategies for all patient demographic groups, irrespective of their socioeconomic status, appears to be a rational approach.

## Figures and Tables

**Table 1.** Comparison of Demographic and Socioeconomic Characteristics Between Compliant and Non-compliant Participants with Follow-up Appointments

**Variables**	**Compliant with Follow-Up Appointments (n = 35)**	**Non-Compliant with Follow-Up Appointments ** **(n = 46)**	*P*
Gender, n (%) Female Male	25 (71.4) 10 (28.6)	37 (80.4) 9 (19.6)	.343
Age, years, median (min-max)	31 (18-76)	30 (18-70)	.620
Educational attainment, n (%) Elementary school Middle school High school Bachelor's degree Master's degree	6 (17.1) 4 (11,4) 19 (54.3) 5 (14.3) 1 (2.9)	11 (23.9) 10 (23.7) 19 (41.3) 6 (13.0) 0 (0.0)	.447
Marital status, n (%) Single Married Divorced	16 (45.7) 18 (52.4) 1 (2.9)	20 (43.5) 23 (50.0) 3 (6.5)	.752
Living arrangement, n (%) Living alone Living with family	4 (11.4) 31 (88.6)	5(10.9) 41(89.1)	1.00
Providing for the family, n (%) Yes No	17 (48.6) 18 (51.4)	22 (47.8) 24 (52.2)	.947
Employment status, n (%) Unemployed Housewife White-collar workers Blue-collar workers Self-employed workers Retired Other	3 (8.6) 2 (5.7) 15 (42.9) 2 (5.7) 5 (14.3) 4 (11.4) 4 (11.4)	6 (13.0) 3 (6.5) 15 (32.6) 0 (0) 10 (21.7) 6 (13.0) 6 (13.0)	.643
Daily working hours, hour/day, median (min-max)	8.0 (0-12)	6.5 (0-12)	.358
Weekly working days, day/week, median (min-max)	5 (0-7)	5 (0-7)	.815
Monthly income, n (%) <10,000 TL 10,000-30,000 TL 30,000-75,000 TL >75,000 TL	9 (25.7) 19 (54.3) 6 (17.1) 1 (2.9)	15 (32.6) 23(50.0) 6(13.0) 2(4.3)	.865
Social security status, n (%) Yes No	33 (94.3) 2 (5.7)	38 (82.6) 8 (17.4)	.174
Alcohol use, n (%) Yes No	13 (37.1) 22 (62.9)	22 (47.8) 24 (52.2)	.336
Tobacco use, n (%) Yes No	20 (57.1) 15 (42.9)	26 (56.5) 20 (43.5)	.955
Vehicle ownership, n (%) Yes No	18 (51.4) 17 (48.6)	22 (47.8) 24 (52.2)	.748
Driver's license status, n (%) Yes No	23 (65.7) 12 (34.3)	31 (67.4) 15 (32.6)	.874
Access to public transportation, n (%) Yes No	34 (97.1) 1 (2.9)	41 (89.1) 5 (10.9)	.228
Method of reaching the hospital, n (%) By foot By public transportation By personal automobile Other	0 (0.0) 19 (45.2) 14 (40.0) 2 (5.7)	1 (2.2) 23 (50.0) 18 (39.1) 4 (8.7)	.783
Distance to hospital from residence, km, median (min-max)	10 (2-250)	10 (1-200)	.792
Distance to hospital from residence, min, median (min-max)	25.0 (5-210)	27.5 (5-280)	.516
Having a caregiver at home, n (%) Yes No	31 (88.6) 4 (11.4)	38 (82.6) 8 (17.4)	.454
Providing care for someone, n% Yes No	6 (17.1) 29 (82.9)	12 (26.1) 34 (73.9)	.337

TL, Turkish Lira.

Statistically significant *P*–value < .05.

**Table 2.** Comparison of Perceived Work Urgency and Injury Impact on Work Ability Between Compliant and Non-compliant Workers

**Variable**	**Compliant with Follow-Up Appointments (n = 24)**	**Non-Compliant with Follow-Up Appointments (n = 28)**	***P***
Perceived urgency of returning to work, n (%) Urgent Somewhat urgent Not urgent	10 (41.7) 5 (20.8) 9 (37.5)	10 (35.7) 7 (25.0) 11 (39.3)	.893
Impact of injury on work ability, n (%) Very important (Impossible to continue) Important (Very difficult) Not very important (Partially continue) Not important (Perform very well)	16 (66.7) 5 (20.8 1 (4.2) 2 (8.3)	15 (53.6) 1 (3.6) 9 (32.1) 3 (10.7)	0.029

Statistically significant *P*-value < .05.

**Table 3.** Comparison of Clinic Characteristics Between Compliant and Non-compliant Participants with Follow-up Appointments

**Variables**	**Compliant with Follow-Up Appointments** **(n = 35)**	**Non-Compliant with Follow-Up Appointments ** **(n = 46)**	*P*
Dominance of injured extremity, n (%) Dominant extremity Non-dominant extremity	21 (60.0) 14 (40.0)	27 (58.7) 19 (41.3)	.906
Work-related accident, n (%) Yes No	7 (20.0) 28 (80.0)	8 (17.4) 38 (82.6)	.765
Nature of injury, n (%) Fracture Tendon injury Nerve injury Tendon + artery injury Tendon + nerve injury Fracture + tendon injury Artery + nerve injury Tendon + artery + nerve injury Fracture + tendon + artery + nerve injury Laceration Crush injury Amputation	9 (25.7) 14 (40.0) 2 (5.7) 1 (2.9) 0 (0.0) 1 (2.9) 1 (2.9) 3 (8.6) 2 (5.7) 0 (0.0) 1 (2.9) 1 (2.9)	12 (26.1) 9 (19.6) 0 (0.0) 2 (4.3) 4 (8.7) 4 (8.7) 1 (2.2) 5 (10.9) 1 (2.2) 1 (2.2) 2 (4.3) 5 (10.9)	.278
MHISS category, n (%) Minor Moderate Severe Major	20 (57.1) 8 (22.9) 1 (2.9) 6 (17.1)	21 (45.7) 8 (17.4) 10 (21.7) 7 (15.2)	.108
BIPQ score, mean (SD)	31.20 (11.56)	30.73 (9.58)	.845
HAD-Anxiety, median (min-max)	7 (0-15)	6 (0-18)	.497
HAD-Anxiety, n (%) ≤10 >10	28 (80.0) 7 (20.0)	39 (84.8) 7 (15.2)	.573
HAD-Depression, median (min-max)	4 (0-11)	4 (0-15)	.305
HAD-Depression, n (%) ≤7 >7	27 (77.1) 8 (22.9)	36 (78.3) 10 (21.7)	.905
Previous injury to the same hand or forearm, n (%) Yes No	9 (25.7) 26 (74.3)	19 (41.3) 27 (58.7)	.144
Having previously undergone physical therapy for an injury, n (%) Yes No	8 (22.9) 27 (77.1)	3 (6.5) 43 (93.5)	.049
EBBS-Benefits, median (min-max)	86 (63-114)	85 (65-115)	.678
EBBS-Barriers, mean (SD)	30.08 (6.77)	29.78 (4.14)	.816
Present rehabilitation program’s implementation process (%) Hospital-based Home-based	21 (60.0) 14 (40.0)	15 (32.6) 31 (67.4)	.014

BIPQ, Brief Illness Perception Questionnaire; EBBS, Exercise Benefits/Barriers Scale; MHISS, Modified Hand Injury Severity Score; SD, standard deviation.

Statistically significant *P*-value < .05.

## Data Availability

The data that support the findings of this study are available on request from the corresponding author.
